# Use of GC-IMS and Stoichiometry to Characterize Flavor Volatiles in Different Parts of Lueyang Black Chicken during Slaughtering and Cutting

**DOI:** 10.3390/foods13121885

**Published:** 2024-06-15

**Authors:** Linlin He, Hui Yang, Fei Lan, Rui Chen, Pengfei Jiang, Wengang Jin

**Affiliations:** 1 College of Biological Science and Engineering, Shaanxi University of Technology, Hanzhong 723001, China; helinlin312@163.com (L.H.); feilan2013@sina.com (F.L.); rchen0411@163.com (R.C.); 2 Shaanxi Province Key Laboratory of Bio-Resources, Shaanxi University of Technology, Hanzhong 723001, China; 3 Qinba Mountain Area Collaborative Innovation Center of Bioresources Comprehensive Development, State Key Laboratory of Biological Resources and Ecological Environment (Incubation), Hanzhong 723001, China; 4 Shaanxi Baisheng Biological Engineering Co., Ltd., Hanzhong 723001, China; 5 College of Food Science and Technology, Dalian Polytechnic University, Dalian 116034, China; jiangpf@dplu.edu.cn

**Keywords:** volatile organic components, Lueyang black chicken, gas chromatography–ion mobility spectroscopy, stoichiometry

## Abstract

Chilled and cut chicken is preferred by consumers for its safeness and readiness to cook. To evaluate the quality characteristics of various chilled chicken products, differences in volatile organic components (VOCs) of six different cut parts (breast, back, leg, heart, liver, and gizzard) of Lueyang black chicken were characterized through gas chromatography–ion mobility spectroscopy (GC-IMS) combined with stoichiometry. A total of 54 peaks in the signal of VOCs were detected by GC-IMS, and 43 VOCs were identified by qualitative analysis. There were 22 aldehydes (20.66–54.07%), 8 ketones (25.74–62.87%), 9 alcohols (4.17–14.69%), 1 ether (0.18–2.22%), 2 esters (0.43–1.54%), and 1 furan (0.13–0.52%), in which aldehydes, ketones, and alcohols were the main categories. Among the six cut parts, the relative content of aldehydes (54.07%) was the highest in the gizzard, and the relative content of ketones (62.87%) was the highest in the heart. Meanwhile, the relative content of alcohols (14.69%) was the highest in the liver. Based on a stable and reliable predictive model established by orthogonal partial least squares–discriminant analysis (OPLS-DA), 3-hydroxy-2-butanone (monomer and dimer), acetone, 2-butanone monomer, hexanal (monomer and dimer), isopentyl alcohol monomer, and n-hexanol monomer were picked out as characteristic VOCs based on variable importance in projection (VIP value > 1.0, *p* < 0.05). Principal component analysis (PCA) and the clustering heatmap indicated that the characteristic VOCs could effectively distinguish the six cut parts of Lueyang black chicken. The specific VOCs responsible for flavor differences among six different cut parts of Lueyang black chicken were hexanal (monomer and dimer) for the gizzard, 2-butanone monomer and hexanal dimer for the breast, hexanal monomer for the back, 3-hydroxy-2-butanone monomer for the leg, 3-hydroxy-2-butanone (monomer and dimer) for the heart, and acetone and isopentyl alcohol monomer for the liver. These findings could reveal references for quality assessment and development of chilled products related to different cut parts of Lueyang black chicken in the future.

## 1. Introduction

Chicken is the favorite choice of meat in the Chinese diet after pork. Among chicken breeds, black chickens have been widely recognized by consumers in China for their nutritional and medicinal benefits [1,2,3]. Lueyang black chicken is a traditional and excellent local breed that is raised freely in Lueyang County, Shaanxi Province, China. It was certified as a geographical indication of agricultural product by the Ministry of Agriculture of China in 2017. This breed exhibits strong disease resistance, rapid growth rate, high protein content, and low fat content, with essential amino acids and trace elements. To date, research on Lueyang black chicken has included studies on its genes [4,5,6], genetic diversity [7], mitochondrial whole genome and molecular phylogeny [8], microsatellite genetic polymorphism analysis [9], muscle transcriptome analysis [10,11], genetic parameters estimation [12], and inosine content determination in muscles [13]. Additionally, there has been research conducted on the volatile aroma of Lueyang black chicken jerky [14]. However, there is still a lack of research on the quality characteristics of Lueyang black chicken.

Flavor is a crucial indicator of the quality of chicken food products, significantly influencing consumer choice and preference [15]. The meat flavor is primarily determined by volatile organic components (VOCs). Previous studies have investigated the flavor profiles of various cut parts from pork [16], sheep [17,18], chicken [19,20], catfish [21], tuna [22,23], and giant salamanders [24]. These studies revealed that the main VOCs varied among different cut parts within the same animal species. For example, Xun et al. [19] and Chen et al. [20] compared VOCs in the leg and breast of Chinese local chickens by GC-MS. In addition to the leg and breast, other cut parts such as the back, heart, liver, and gizzard are also popular among consumers due to their unique nutritional value and distinct flavors. However, there is currently a lack of research on flavor components in different cut parts of chicken.

Although GC-MS is the widely used method for VOCs analysis, its sample preparation is very complex and time-consuming. Recently, GC-IMS has emerged as a powerful tool for separating and detecting VOCs in food. Compared to GC-MS, GC-IMS offers simple sample preparation, high sensitivity, high resolution, high analysis efficiency, and visualization of VOCs [25,26]. Up to now, The GC-IMS technology has been broadly utilized for the analysis of VOCs in different cut parts of various edible animals, such as the loin, shoulder, ham, and belly of pork [16]; the longissimus dorsi, biceps femoris, and triceps brachii of Tan sheep [18]; the dorsal and abdominal muscles of yellowfin tuna, bigeye tuna, and bluefin tuna [22]; the dorsal and abdominal muscles of *Thunnus thynnus* [23]; and the head, claw, back, tail, abdomen, and liver of giant salamander [24]. These studies proved that the GC-IMS technology was more superior to GC-MS for VOCs analysis in terms of rapidness, easiness, and visualization, etc.

Our previous studies optimized the processing conditions of Lueyang black chicken sausage by a single factor and orthogonal test [27] and analyzed the nutritional content and antioxidant effects of the soup made from Lueyang black chicken on aging mice induced by D-galactose [28]. As the chilled chicken cut products are popular, it is very important to characterize VOCs in different cut portions of Lueyang black chicken. In this study, the VOCs in the breast, back, leg, heart, liver, and gizzard of Lueyang black chicken were measured by GC-IMS, and characteristic VOCs of the six different cut parts were screened out through qualitative analysis combined with stoichiometry, which can provide references for future quality control and the development of chilled products related to different cuts from Lueyang black chicken.

## 2. Materials and Methods

### 2.1. Materials and Chemicals

Three 8-month-old, fresh Lueyang black chicken roosters, weighing (2.42 ± 0.43) kg each, were procured from Black Phoenix Black Chicken Breeding Co., Ltd. at Lueyang County (Hanzhong, China). Following the slaughter of the chickens, six different cut parts of Lueyang black chicken (breast, back, leg, heart, liver, and gizzard) were taken out and transported to the laboratory on ice. Analytical-grade 2-butanone, 2-pentanone, 2-hexanone, 2-heptanone, 2-octanone, and 2-nonanone were supplied by Guoyao Reagent Co., Ltd. (Beijing, China).

### 2.2. Preparation of Six Different Cutting Parts of Lueyang Black Chicken

The six chicken cuts mentioned above were crushed and homogenized with a tissue masher homogenizer (JJ-2B, Changzhou, China) for VOCs analysis.

### 2.3. GC-IMS Assay of VOCs in Six Different Cut Parts of Lueyang Black Chicken

The VOCs in six different cuts of the Lueyang black chicken were detected with GC-IMS. Each kind of cut parts was measured three times using 2.0 g of the cut part placed in a 20.0 mL headspace bottle. The samples were then injected with 500.0 μL headspace gas nitrogen (purity ≥ 99.99%) into the injector after incubating at 60℃ for 15 min and then detected by a GC-IMS instrument (FlavourSpec^®^, Dortmund, Germany) [24,29]. The GC separation was performed on the MXT-5 column at 60℃ with nitrogen (purity ≥ 99.99%) as a carrier gas for 20 min. The start-up gas flow rate was set at 2.0 mL/min and maintained for 2 min before linearly enlarged to 10 mL/min within 10 min and then further expanded to 100 mL/min within 20 min. The IMS was conducted with a 45℃ IMS detector and 150 mL/min nitrogen (purity ≥ 99.99%) flow rate and analyzed for 30 min. Before analyzing the sample, the calibration solution of the six C4–C9 n-acetones mentioned above (the retention index was set to be 100 times its carbon number) was tested under the same gas phase conditions as the sample, and calibration curves of retention index (RI) (known) and retention time (RT) were established. The ketones mentioned above served as immigrant markers to determine the relative ratio of VOCs in different cuts based on drift time (DT) and retention index (RI) data provided by the instrument’s fragment libraries: the IMS drift time database and NIST 2014. The peak volume of each identified compound was also calculated to show its relative content.

### 2.4. Statistical Analysis

The VOCs were qualitatively identified using the NIST 2014 software and a self-built IMS database. The Reporter plugin facilitated direct comparison of spectral differences, while the Gallery Plot plugin allowed for quantitative comparison of VOCs differences between samples through fingerprint spectrum analysis. Excel 2010 was used to create bar graphs illustrating relative content changes of different components. Principal component analysis (PCA) as well as the establishment and validation of orthogonal partial least squares–discriminant analysis (OPLS-DA) modeling were conducted by SIMCA-P 14.1 software. The clustering heatmap was generated using the BioDeep tool assistant (https://www.biodeep.cn/home/tool) (accessed on 30 April 2024).

## 3. Results and Discussion

### 3.1. GC-IMS 3D and 2D Spectrum of VOCs in Six Cut Parts of Lueyang Black Chicken

**Figure 1:**
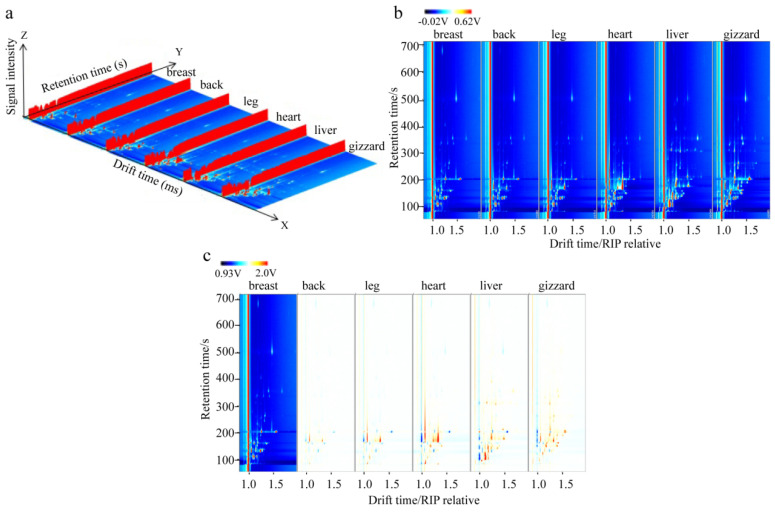
The GC−IMS spectra of six different cuts of Lueyang black chicken. (**a**) Three−dimensional spectra. (**b**) Two−dimensional top-view spectra. (**c**) Two−dimensional difference spectra.

The VOCs in the six cuts of Lueyang black chicken were detected using a GC-IMS instrument. The 3D spectra of the VOCs in these cut parts were obtained (Figure 1a) with the DT, ion relative retention time (RT), and signal intensity represented on the *X*, *Y*, and *Z* axes, respectively. The signal intensity showed the amplitude of the peak [30,31]. However, due to the relatively similar nature of the 3D spectra for all six different cut parts, it was difficult to visually compare their differences. Further dimensionality reduction was needed to facilitate this comparison [32,33].

To provide a more intuitive comparison of the variations in VOCs among the six different cutting parts, 2D view spectra (Figure 1b) were derived from the original 3D spectra in Figure 1a. Additionally, 2D difference spectra (Figure 1c) were obtained by deducting the breast spectrum as a base. These visual representations showed that different VOCs exhibited varying horizontal migration time and vertical RT values across different samples. Furthermore, the characteristic spectra obtained from GC-IMS showed noticeable differences among all six different cut parts of the Lueyang black chicken (Figure 1b,c), which might be related to the differences in their nutritional and chemical compositions.

### 3.2. Fingerprint of VOCs in Six Different Cut Parts of Lueyang Black Chicken

**Figure 2:**
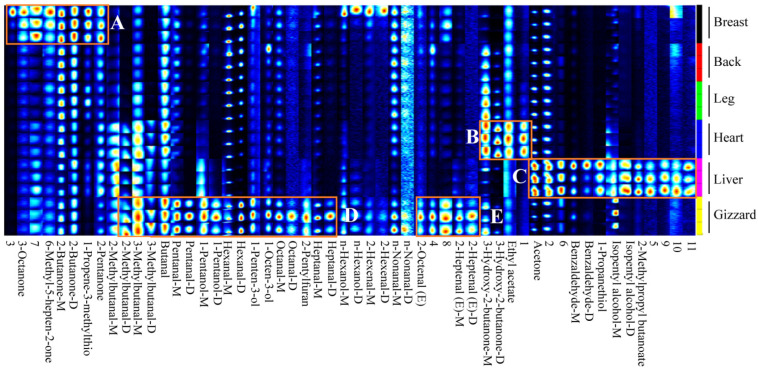
Gallery fingerprint of the VOCs of six different cut parts of Lueyang black chicken. Compounds with suffix D and M represent dimer and monomer, respectively. A, B, and C refer to the region with higher levels of VOCs in the breast, heart, and liver in the gallery fingerprint, respectively. D and E refer to the regions with higher levels of VOCs in the gizzard in the gallery fingerprint.

A fingerprint was built to display the VOCs differences in the six different cuts through three parallel tests (Figure 2). The horizontal direction represents various VOCs found within each cut part (breast, back, leg, heart, liver, and gizzard) from top to bottom, while the vertical direction represents identical VOCs across all six different cuts. The M and D behind the same VOCs refer to the monomer and dimer of this compound, respectively. They are the same compound with the same CAS number and molecular weight, yet they have different DT, as shown in Table 1.

As demonstrated in Figure 2, there are significant differences in the VOCs profiles among all six types of Lueyang black chicken cuts. Specifically, region A displayed relatively higher levels of certain compounds, namely 6-methyl-5-hepten-2-one, 3-octanone, 2-butanone (D and M), 1-propene-3-methylthio, 2-pentanone, unknown compound 3, and unknown compound 7 in chicken breast compared to lower levels observed in chicken back, leg, heart, liver, and gizzard. The VOCs in region B, including 3-hydroxy-2-butanone (M and D), ethyl acetate, and unknown compound 1, were found to be relatively higher in chicken heart but relatively low in chicken breast, back, leg, liver, and gizzard. In region C, the VOCs including acetone, benzaldehyde (M and D), 1-propanethiol, isopentyl alcohol (M and D), 2-methylpropyl butanoate, and unknown compounds 2, 5, 6, 9, 10, and 11 were relatively higher in chicken liver but lower in chicken breast, back, leg, heart, and gizzard. Region D contained VOCs such as 2-methylbutanal (D), 3-methyl butanal (D and M), butanal, pentanal (D and M), 1-pentanol (D and M), hexanal (D and M), 1-penten-3-ol, 1-octen-3-ol, octanal (D and M), 2-pentylfuran, and heptanal (D and M). The VOCs identified in region E included 2-octenal (E), 2-heptenal (E) (D and M), and unknown compounds 4 and 8. The peak volume of VOCs in regions D and E was relatively higher in the gizzard and lower in the breast, back, leg, heart, and liver. These identified VOCs may be the major promoters for the flavors of different cuts of Lueyang black chicken. However, due to the limitations of GC-IMS analysis [16,34], the present study only identified 43 VOCs from a total of 54 signal peaks through GC-IMS library, indicating that additional detection techniques (e.g., GC-MS and GC-O) should be performed to identify the unknown VOCs and provide a more complete flavor profile in future. Similar studies of VOCs analysis using GC-IMS were also reported by previous publications [15,31].

### 3.3. Qualitative Analysis of VOCs in Six Different Cut Parts of Lueyang Black Chicken

**Figure 3:**
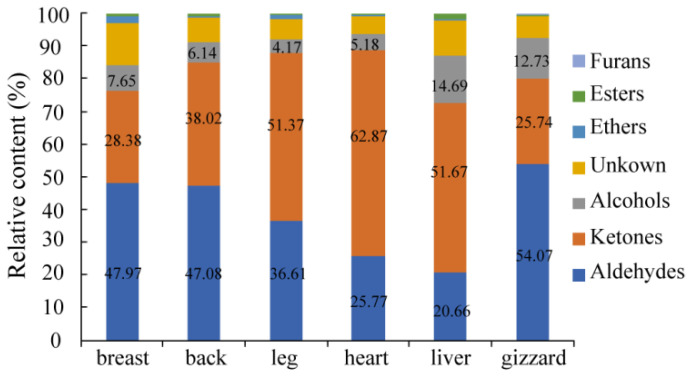
The relative content changes of the VOCs of six different cut parts of Lueyang black chicken.

VOCs were qualitatively analyzed according to the DT and RT of IMS. The chemicals, CAS numbers, molecular weight (MW), RI, DT, and compound peak volumes for various VOCs are shown in Table 1. The relative contents of the VOCs in six different chicken cuts are shown in Figure 3. There are many types of VOCs in chickens, mainly including aldehydes, ketones, alcohols, esters, and ethers [19,32,35,36]. A total of 54 signal peaks were obtained from six different cut parts of Lueyang black chicken, from which 43 VOCs were identified, including 22 aldehydes (20.66–54.07%), 8 ketones (25.74–62.87%), 9 alcohols (4.17–14.69%), 1 ether (0.18–2.22%), 2 esters (0.43–1.54%), and 1 furan (0.13–0.52%) (Table 1 and Figure 3). The primary VOCs found in the six different cuts of Lueyang black chicken consist of ketones, aldehydes, and alcohols, accounting for 83.99–93.81% of the total VOCs. Similar studies have indicated that ketones, aldehydes, and alcohols are also the main VOCs present in the breast and leg of other Chinese local chicken breeds such as Chinese Piao chicken and Yanjin silky fowl [19].

Aldehydes are known to have a relatively low threshold, which is the main contributor to the chicken meat aroma [36,37]. The highest aldehydes content was found in the gizzard (54.07%), followed closely by the back (47.08%) and breast (47.97%). In contrast, lower levels of aldehydes were observed in the heart (25.77%) and liver (20.66%) (Figure 3). Notably, compared to Zhanjiang Sanhuang chicken, Qingyuan partridge chicken, and fast-grown chicken, Lueyang black chicken exhibited lower levels of aldehydes at 47.97% in the breast and 36.61% in the leg [20]. Hexanal was identified as having the highest peak volume among all aldehydes present in these cuts mentioned above. The peak volume of hexanal in the gizzard was the highest, followed by the back, breast, leg, heart, and liver (Table 1), which can endow these above-mentioned cuts with aromas of grass, tallow, and fat. The total aldehyde content was the highest in VOCs, and the content of hexanal was the highest among aldehydes in the breast of Jingxing yellow chicken, Tian-nong partridge chicken, and Wenchang chicken [37,38]. The peak volumes of 2-heptenal (E) (D), n-nonanal-M, butanal, heptanal-M, 3-methyl butanal-M, and pentanal-M were larger among aldehydes, which indicated that the content of these VOCs was higher among aldehydes. Furthermore, it is worth noting that free-range chickens like Lueyang black chickens tend to have higher hexanal content compared to cage-raised chickens—a factor that may contribute to their unique aroma profile [39]. Research has also shown a close relationship between *SLC27A1* gene expression and peroxisome proliferator-activated receptor (PPAR) pathway activation with hexal content in Chinese local chicken VOCs [40]. Yuan et al. [41] identified 16 candidate genes related to fatty acid pathways while highlighting a significant association between polyunsaturated fatty acids (PUFAs) and both aldehyde content as well as hexanal content within Chinese local chickens. The peak volume of 2-heptenal (E) (D) was found to be higher among aldehydes, imparting the different cuts of Lueyang black chicken with aromas of soap, fat, and almond.

In terms of ketones, The highest peak volume was observed in the heart (62.87%), followed closely by the liver (51.67%) and leg (51.37%). The back, breast, and gizzard had much lower peak volumes of ketones at 38.02%, 28.38%, and 25.74%, respectively, compared to the heart, liver, and leg. Additionally, the content of ketones in the breast (28.38%) was lower than that in the back (38.02%), while it was higher than that in the gizzard (25.74%) (Figure 3). Among ketones, acetone, 3-hydroxy-2-butanone (M and D), 2-pentanone, and 2-butanone-M showed larger peak volumes, indicating higher contents of these VOCs among ketones. Furthermore, the peak volume of acetone was the highest among ketones in the liver, followed by the leg, heart, gizzard, back, and breast (Table 1), which can endow the different cuts mentioned above with apple and pear aromas. The peak volume of 3-hydroxy-2-butanone-D was also found to be higher among ketones in the heart, followed by the leg, gizzard, back, liver, and breast (Table 1), which can endow the different cuts mentioned above with butter and cream aromas.

In terms of alcohols content, the liver had the highest percentage (14.69%), followed by the gizzard, breast, back, heart, and leg (12.73%, 7.65%, 6.14%, 5.18%, and 4.17%, respectively). The content of alcohols in the breast and leg of Lueyang black chicken accounted for 7.65% and 4.17%, respectively (Figure 3). These percentages are much higher than those of Zhanjiang Sanhuang chicken, Qingyuan partridge chicken, and fast-grown chicken [20]. The differences in the content of aldehydes, ketones, and alcohols may be due to different factors such as breed, age, diet of chicken, detection methods, and so on.

### 3.4. OPLS-DA and Model Validation

**Figure 4:**
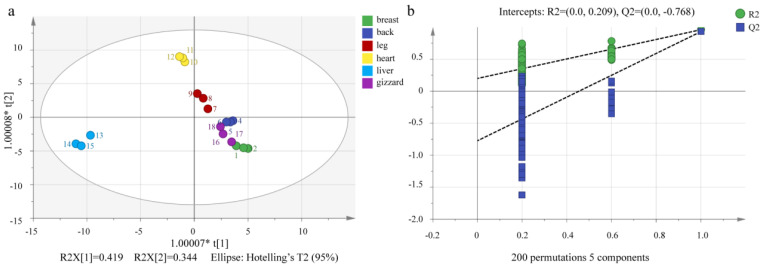
OPLS−DA scores of VOCs of six different chicken cuts (**a**) and displacement test (**b**).

The common stoichiometric methods include OPLS-DA, PCA, artificial neural network, genetic algorithm, and so on. The stoichiometric methods used in this study were OPLS-DA, PCA, and clustering heatmap. OPLS-DA, unlike PCA, is a method of statistics for supervising discriminant analysis. It establishes a relationship model between material expression and sample categories to achieve the prediction of sample categories. *R*^2^*Y* and *R*^2^*X* represent the model’s explanatory rate for the *Y* and *X* matrices, respectively, while *Q^2^* represents the model’s predictive ability. As shown in Figure 4a, it is evident that the different cuts of the Lueyang black chicken (breast, back, leg, heart, liver, and gizzard) are well dispersed on the OPLS-DA score map, with a very close effect on the PCA scatter plot. The reliability of the model was verified to ensure against over-fitting by using a permutation test. Parameters of *Q*^2^ and *R*^2^ greater than 0.5 but less than 1.0 are considered more accurate [42,43]. Most information on VOCs in the six different cut parts was covered by this model with *Q*^2^ (cum) = 0.848, *R*^2^*X*(cum) = 0.975, and *R*^2^*Y*(cum) = 0.921. Most cut samples of Lueyang black chicken could be classified by the OPLS-DA map (Figure 4a). Figure 4b shows the correlation between *R^2^*/*Q*^2^ from the permutation test and the original data *R*^2^/*QC*. The regression line for *Q*^2^ (−0.768) is less than zero at the crossing point after 200 cross-validations, and both *R*^2^ and *Q*^2^ are below the raw values in all tests, suggesting that the established OPLS-DA model is not over-fitting but stable and reliable [30,31,34,43].

### 3.5. Screening of Characteristic VOCs in Six Different Cut Parts of Lueyang Black Chicken

**Figure 5:**
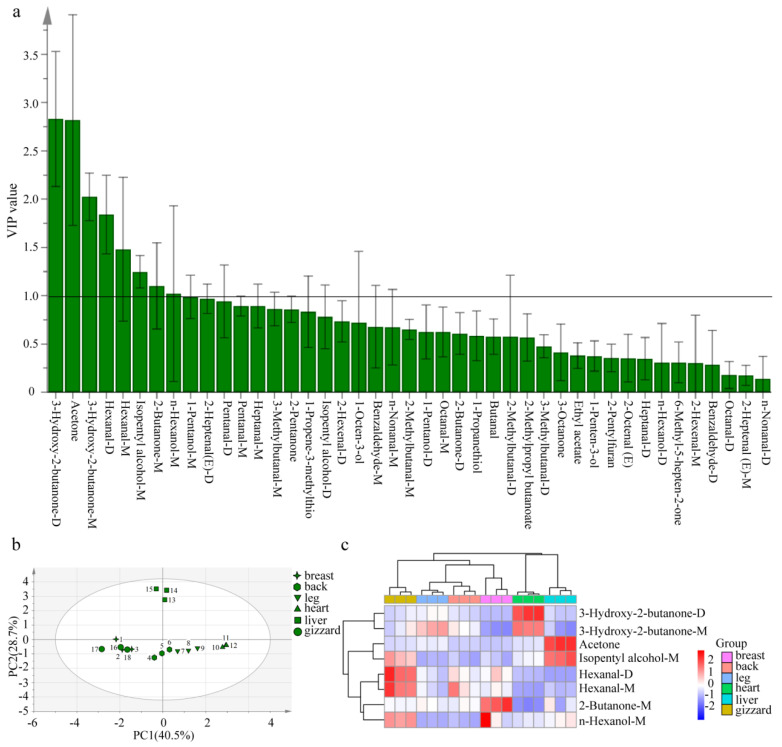
Screening of characteristic VOCs in different cutting parts of Lueyang black chicken (**a**) VIP value; (**b**) principal component score map; (**c**) clustering heatmap. Compounds with suffix −D and −M represent dimer and monomer, respectively.

According to VIP values in the OPLS-DA model, the contribution of each variable to the classification can be quantified. Characteristic VOCs were screened based on VIP > 1.0 and *p* < 0.05. This screening method has been used in various types of food to select characteristic flavor components [30,31,43,44,45]. In this study, 8 characteristic VOCs were selected from 43 VOCs (as shown in Figure 5a) in six different cuts of Lueyang black chicken. These include four ketones (3-hydroxy-2-butanone (M and D), acetone, and 2-butanone-M), two aldehydes (hexanal (M and D)), and two alcohols (isopentyl alcohol-M and n-hexanol-M). Among the eight characteristic VOCs, 3-hydroxy-2-butanone-D exhibited the highest VIP value, with aromas of butter and cream. This was followed by acetone (with aromas of apple and pear), 3-hydroxy-2-butanone-M (with aromas of butter and cream), hexanal (D and M) (with aromas of grass, tallow, and fat), isopentyl alcohol-M (with whiskey, malt, and burnt aromas), 2-butanone-M (with aromas of ether), and n-hexanol-M (with resin, flower, and green aromas).

The compound peak volume analysis of the eight characteristic VOCs revealed that among the six different cuts of Lueyang black chicken, the compound peak volume of 3-hydroxy-2-butanone (M and D) was highest in the heart, which was observed in Lueyang black chicken. Additionally, the compound peak volume of acetone was found to be highest in the liver, while hexanal (D and M) showed the highest compound peak volume in the gizzard, and both compounds were found in Lueyang black chicken. Furthermore, it was observed that 2-butanone-M exhibited the highest compound peak volume in the breast of Lueyang black chicken (Table 1). It is worth noting that among these eight VOCs identified in six different cut parts of Lueyang black chicken, 3-hydroxy-2-butanone was not found in the breast VOCs of Jingxing yellow chicken, Tian-nong partridge chicken, and Wenchang chicken [37,38]; the thigh meat of three kinds of Korean native chicken (Hanhyup No. 3, Woorimatdag No. 1, and Woorimatdag No. 2) and commercial broiler chicken [46]; and the breast and leg of Yanjin silky fowl, Piao chicken, Qingyuan partridge chicken, fast-grown chicken, and Zhanjiang Sanhuang chicken [19,20], making it a novel finding. Acetone emerged as the main VOC in the leg and breast of Yanjin silky fowl and Piao chicken [19], whereas hexanal took precedence in the leg and breast of Qingyuan partridge chicken, fast-grown chicken, Zhanjiang Sanhuang chicken, and Piao chicken [19,20].

PCA and clustering analysis were performed on these eight selected VOCs (Figure 5b,c). PCA results revealed that the cumulative contribution ratio for these characteristic VOCs was 69.2% (with PC1 40.5% and PC2 28.7%) (Figure 5b), effectively explaining differences across most samples. In addition, the eight characteristic VOCs in the same cuts of Lueyang black chicken were relatively concentrated, which could better distinguish the different cuts of Lueyang black chicken. Based on the signal intensities derived from these VOCs, a clustering heatmap (Figure 5c) was generated to illustrate differences in the eight characteristic VOCs in the six different cuts of the Lueyang black chicken. This analysis categorized flavor characteristics into three groups. The first group pertains to the gizzard; the second group includes the leg, back, and breast; and the third group encompasses the heart and liver. (Figure 5c). Among the eight characteristic VOCs, specific substances were identified as having a significant impact on the flavor of different cutting parts of Lueyang black chicken. These include hexanal (D and M) for the gizzard, 2-butanone-M and hexanal-D for the breast, hexanal M for the back, 3-hydroxy-2-butanone-M for the leg, 3-hydroxy-2-butanone (D and M) for the heart, and acetone and isopentyl alcohol-M for the liver (Figure 5c). Jin et al. previously utilized OPLS-DA combined with VIP > 1.0 and *p* < 0.05 to identify 22 differentially VOCs from cooked wheat grains with three colors [30] as well as to screen 19 differentially VOCs from pigmented rice with five various colors after puffing [31]. In this study, the eight potential characteristic VOCs were screened from six different cut parts of Lueyang black chicken by GC-IMS and the OPLS-DA model, with VIP > 1.0 and *p* < 0.05. However, quantitative analysis was still lacking, and the sample size of the present study was small. Future studies should include a larger sample size to validate the reliability and generalizability of these characteristic VOCs. Moreover, the present study only used the emerging GC-IMS technology for VOCs analysis. Several reports pointed out that both GC-IMS and GC-MS can be used in complementary ways for VOCs analysis [35,37,43]. So, it is recommended that future studies should combine GC-MS, GC-O, and relative odor activity values to further reveal the detailed variations of VOCs from different cuts of Lueyang black chicken.

## 4. Conclusions

In summary, a fingerprint map of VOCs in the six different cut parts of Lueyang black chicken was established using GC-IMS technology. There were 43 flavor volatiles identified, primarily consisting of aldehydes, ketones, and alcohols. A stable and reliable OPLS-DA model was successfully established, and eight characteristic VOCs were screened, including four ketones, two aldehydes, and two alcohols. Among them, the important VOCs affecting the six different cut flavor profiles of Lueyang black chicken were determined to be 3-hydroxy-2-butanone (D and M), acetone, and hexanal-D (VIP > 1.5). The specific VOCs responsible for flavor differences among the breast, back, leg, heart, liver, and gizzard of Lueyang black chicken were as follows: hexanal (D and M) for the gizzard, 2-butanone-M and hexanal-D for the breast, hexanal-M for the back, 3-hydroxy-2-butanone-M for the leg, 3-hydroxy-2-butanone (M and D) for the heart, and acetone and isopentyl alcohol-M for the liver. Furthermore, the PCA and clustering analysis of the eight characteristic VOCs indicated that they could effectively distinguish between the different cuts of Lueyang black chicken. However, the weaknesses of this study were its small sample size and the lacking accuracy of the quantitative analysis. Detailed information regarding the validation of larger sample scale, GC-MS, and GC-O of different Lueyang black chicken cuts will be reported elsewhere.

## Figures and Tables

**Table 1:** Qualitative analysis of VOCs in six different cutting parts of Lueyang black chicken.

NO	Chemicals	CAS	MW	RI	DT	Compound Peak Volume					
						Breast	Back	Leg	Heart	Liver	Gizzard
Aldehydes	2-Heptenal (E)-D	18829-55-5	112.2	949.2	1.66725	2101.08 ± 22.17 a	1684.53 ± 26.00 b	1642.28 ± 21.71 b	1354.58 ± 46.98 c	877.55 ± 86.66 d	1350.21 ± 30.44 c
	2-Heptenal (E)-M	18829-55-5	112.2	949.2	1.25632	14.13 ± 0.92 b	14.49 ± 0.44 b	15.37 ± 4.39 b	15.17 ± 2.64 b	20.85 ± 7.66 b	45.31 ± 15.51 a
	2-Hexenal-D	505-57-7	98.1	840.8	1.51179	93.59 ± 21.99 c	183.91 ± 61.75 bc	113.47 ± 10.70 bc	129.74 ± 11.60 bc	214.40 ± 21.82 b	700.60 ± 129.69 a
	2-Hexenal-M	505-57-7	98.1	846.8	1.18058	42.82 ± 42.78 a	13.25 ± 1.33 a	15.75 ± 4.79 a	14.03 ± 2.98 a	15.52 ± 3.12 a	27.73 ± 2.98 a
	2-Methylbutanal-D	96-17-3	86.1	651.9	1.40071	184.42 ± 116.42 a	61.64 ± 1.80 bc	57.72 ± 2.09 bc	51.07 ± 1.00 bc	38.15 ± 1.13 c	138.90 ± 19.68 ab
	2-Methylbutanal-M	96-17-3	86.1	658.1	1.16897	121.66 ± 11.91 d	133.08 ± 7.26 d	191.25 ± 24.08 c	433.86 ± 17.65 b	515.12 ± 28.65 a	416.27 ± 52.15 b
	2-Octenal (E)	2548-87-0	126.2	1053.9	1.33121	123.60 ± 19.64 ab	107.28 ± 18.62 b	103.10 ± 1.75 b	106.33 ± 2.22 b	117.25 ± 15.90 b	232.69 ± 69.26 a
	3-Methylbutanal-D	590-86-3	86.1	642.4	1.40677	10.26 ± 1.54 c	9.83 ± 1.64 c	12.54 ± 2.35 c	131.85 ± 21.37 b	143.68 ± 21.63 b	206.53 ± 73.94 a
	3-Methylbutanal-M	590-86-3	86.1	641.8	1.18336	279.74 ± 49.75 e	278.84 ± 24.42 e	418.41 ± 64.73 d	708.97 ± 15.27 b	578.42 ± 23.92 c	867.60 ± 39.29 a
	Benzaldehyde-D	100-52-7	106.1	953.6	1.47255	25.92 ± 5.04 b	27.88 ± 1.19 b	26.66 ± 3.69 b	31.25 ± 2.71 b	168.92 ± 107.95 a	33.64 ± 6.52 b
	Benzaldehyde-M	100-52-7	106.1	953.7	1.14842	119.07 ± 37.24 b	101.53 ± 37.28 b	77.28 ± 8.63 b	98.16 ± 3.59 b	765.29 ± 252.30 a	242.21 ± 122.42 b
	Butanal	123-72-8	72.1	590.3	1.29064	558.53 ± 12.90 d	655.50 ± 2.88 c	658.49 ± 22.70 c	766.35 ± 7.97 b	517.43 ± 36.31 e	926.23 ± 23.78 a
	Heptanal-D	111-71-7	114.2	894.5	1.69931	29.5 ± 7.58 b	24.45 ± 4.15 b	25.04 ± 3.93 b	20.40 ± 0.35 b	19.01 ± 2.54 b	139.96 ± 67.37 a
	Heptanal-M	111-71-7	114.2	895.0	1.33916	559.89 ± 219.41 b	345.31 ± 50.07 c	289.05 ± 40.16 c	337.73 ± 15.96 c	251.79 ± 30.67 c	903.13 ± 163.29 a
	Hexanal-D	66-25-1	100.2	792.6	1.56223	2370.05 ± 621.36 b	2149.47 ± 569.14 bc	1364.67 ± 424.77 cd	804.37 ± 61.08 de	358.08 ± 18.12 e	5011.03 ± 700.60 a
	Hexanal-M	66-25-1	100.2	797.2	1.26376	1908.28 ± 146.79 bc	2358.59 ± 553.62 b	1647.73 ± 211.59 cd	1264.20 ± 38.83 d	1479.86 ± 59.65 cd	3141.39 ± 299.53 a
	n-Nonanal-D	124-19-6	142.2	1104.5	1.94640	84.08 ± 6.42 a	79.23 ± 18.35 a	76.32 ± 12.59 a	74.06 ± 11.98 a	66.67 ± 8.02 a	87.99 ± 14.76 a
	n-Nonanal-M	124-19-6	142.2	1104.1	1.47787	1005.99 ± 39.10 a	930.52 ± 134.98 ab	833.57 ± 113.48 ab	701.62 ± 84.54 b	341.10 ± 91.85 c	978.79 ± 260.68 a
	Octanal-D	124-13-0	128.2	1000.1	1.82369	32.24 ± 4.59 b	26.24 ± 3.83 b	25.74 ± 3.76 b	22.86 ± 2.40 b	23.62 ± 4.25 b	55.41 ± 22.61 a
	Octanal-M	124-13-0	128.2	1000.4	1.40732	368.11 ± 50.40 b	326.61 ± 47.88 b	277.18 ± 9.09 b	235.26 ± 21.77 bc	123.60 ± 20.32 c	640.55 ± 178.66 a
	Pentanal-D	110-62-3	86.1	694.0	1.42362	71.44 ± 32.18 b	63.79 ± 17.68 b	53.72 ± 17.65 b	69.03 ± 9.86 b	27.94 ± 2.31 b	942.41 ± 300.10 a
	Pentanal-M	110-62-3	86.1	687.6	1.19251	484.50 ± 111.72 b	544.99 ± 54.24 b	532.34 ± 73.77 b	505.19 ± 16.14 b	244.05 ± 17.20 c	1253.77 ± 85.32 a
Ketones	2-Butanone-D	78-93-3	72.1	575.7	1.24613	20.72 ± 3.28 d	22.84 ± 3.95 d	35.46 ± 8.62 d	229.45 ± 28.71 b	142.87 ± 20.91 c	348.04 ± 93.01 a
	2-Butanone-M	78-93-3	72.1	568.7	1.06064	2286.45 ± 139.52 a	1506.66 ± 70.91 b	1520.11 ± 75.23 b	1043.18 ± 36.02 c	1411.16 ± 259.43 b	1495.13 ± 11.54 b
	2-Pentanone	107-87-9	86.1	678.1	1.12126	594.20 ± 44.87 a	357.83 ± 20.01 c	252.64 ± 3.48 d	324.93 ± 2.86 c	415.66 ± 46.30 b	221.10 ± 18.54 d
	3-Hydroxy-2-butanone-D	513-86-0	88.1	711.7	1.33104	409.61 ± 57.72 d	1537.15 ± 385.08 c	2933.70 ± 527.46 b	10,178.36 ± 766.26 a	975.80 ± 481.85 cd	1709.35 ± 587.25 c
	3-Hydroxy-2-butanone-M	513-86-0	88.1	710.0	1.06522	667.43 ± 156.26 d	2268.30 ± 277.17 c	3390.50 ± 321.86 b	4117.34 ± 87.29 a	913.74 ± 524.11 d	1972.62 ± 520.37 c
	3-Octanone	106-68-3	128.2	980.0	1.30484	146.76 ± 38.88 a	61.35 ± 8.14 b	53.41 ± 3.85 b	44.51 ± 1.53 b	54.07 ± 6.62 b	46.97 ± 5.34 b
	6-Methyl-5-hepten-2-one	110-93-0	126.2	983.5	1.17573	75.22 ± 18.94 a	31.84 ± 5.57 b	40.59 ± 2.47 b	34.37 ± 4.22 b	25.89 ± 7.07 b	33.61 ± 6.01 b
	Acetone	67-64-1	58.1	484.9	1.11614	2064.86 ± 143.50 e	2387.14 ± 213.40 de	3641.07 ± 100.37 b	3243.05 ± 51.70 bc	13,343.97 ± 607.55 a	2907.22 ± 329.76 cd
Alcohols	1-Octen-3-ol	3391-86-4	128.2	974.3	1.15835	130.42 ± 19.71 b	230.37 ± 148.17 ab	116.70 ± 15.49 b	157.23 ± 19.48 b	177.56 ± 13.26 b	328.79 ± 71.90 a
	1-Pentanol-D	71-41-0	88.1	761.3	1.51238	34.48 ± 9.77 b	33.67 ± 15.33 b	24.33 ± 4.33 b	22.06 ± 1.51 b	72.08 ± 13.85 b	434.61 ± 138.53 a
	1-Pentanol-M	71-41-0	88.1	761.1	1.25622	327.62 ± 68.58 cd	353.35 ± 146.82 c	175.13 ± 34.18 cd	156.72 ± 6.83 d	841.35 ± 40.33 b	1246.36 ± 174.73 a
	1-Penten-3-ol	616-25-1	86.1	669.7	0.94040	106.34 ± 37.62 b	63.34 ± 4.77 c	68.70 ± 5.32 c	64.64 ± 3.80 c	78.04 ± 6.02 bc	147.41 ± 11.06 a
	1-Propanethiol	107-03-9	76.2	616.7	1.36762	23.31 ± 2.52 b	23.44 ± 3.58 b	24.55 ± 4.50 b	28.72 ± 3.71 b	491.52 ± 60.55 a	32.32 ± 1.34 b
	Isopentyl alcohol-D	123-51-3	88.1	727.7	1.49232	40.75 ± 13.95 b	40.00 ± 1.48 b	33.99 ± 1.82 b	45.65 ± 1.82 b	843.70 ± 92.10 a	59.11 ± 7.37 b
	Isopentyl alcohol-M	123-51-3	88.1	727.5	1.24756	500.44 ± 182.18 d	428.78 ± 154.43 d	387.14 ± 31.15 d	852.17 ± 41.41 c	2057.37 ± 125.11 a	1483.22 ± 184.54 b
	n-Hexanol-D	111-27-3	102.2	859.2	1.64002	43.20 ± 45.09 a	11.30 ± 1.55 a	12.22 ± 2.56 a	10.63 ± 0.59 a	19.39 ± 3.38 a	39.70 ± 1.60 a
	n-Hexanol-M	111-27-3	102.2	861.8	1.32879	481.55 ± 362.20 ab	135.64 ± 26.31 c	120.08 ± 6.50 c	245.13 ± 19.16 bc	333.80 ± 46.80 abc	547.46 ± 18.58 a
Ethers	1-Propene-3-methylthio	10152-76-8	88.2	691.0	1.04401	489.94 ± 58.67 a	146.30 ± 11.11 cd	251.30 ± 66.91 b	81.51 ± 2.30 de	181.55 ± 6.39 c	62.66 ± 15.29 e
Esters	2-Methylpropyl butanoate	539-90-2	144.2	948.4	1.32934	98.11 ± 58.69 b	55.81 ± 0.83 b	44.89 ± 3.05 b	62.58 ± 9.13 b	469.63 ± 74.85 a	112.11 ± 4.46 b
	Ethyl acetate	141-78-6	88.1	603.1	1.09543	74.29 ± 5.78 b	71.69 ± 11.92 b	54.83 ± 3.38 c	120.71 ± 6.88 a	44.09 ± 2.71 c	45.39 ± 3.88 c
Furans	2-Pentylfuran	3777-69-3	138.2	987.1	1.25468	40.29 ± 1.43 b	66.30 ± 18.45 b	45.42 ± 6.82 b	38.96 ± 3.21 b	53.69 ± 5.39 b	177.37 ± 42.15 a

Compounds with suffix D and M represent dimer and monomer, respectively. Different letters in the same line indicate a significant difference (*p* < 0.05).

## Data Availability

The original contributions presented in the study are included in the article, further inquiries can be directed to the corresponding author.
